# Early initiation of second-line therapy in primary immune thrombocytopenia: insights from real-world evidence

**DOI:** 10.1007/s00277-023-05289-0

**Published:** 2023-06-10

**Authors:** Adam Cuker, Brian Buckley, Marie-Catherine Mousseau, Aditya Anand Barve, Jens Haenig, James B. Bussel

**Affiliations:** 1 Department of Medicine and Department of Pathology and Laboratory Medicine, Perelman School of Medicine, Hospital of the University of Pennsylvania, University of Pennsylvania, 3 Dulles, 3400 Spruce Street, Philadelphia, PA USA; 2 Novartis Ireland Limited, Dublin, Ireland; 3 Novartis Pharma AG, Basel, Switzerland; 4 Department of Pediatrics, Weill Cornell Medicine, New York, NY USA

**Keywords:** Primary immune thrombocytopenia, Second-line treatment, Corticosteroid toxicity, Eltrombopag, Romiplostim, Rituximab

## Abstract

To compare patients with primary immune thrombocytopenia (ITP) prescribed early (within 3 months of initial ITP treatment) second-line treatment (eltrombopag, romiplostim, rituximab, immunosuppressive agents, splenectomy) with or without concomitant first-line therapy to those who received only first-line therapy. This real-world retrospective cohort study of 8268 patients with primary ITP from a large US-based database (Optum^® ^de-identified Electronic Health Record [EHR] dataset) combined electronic claims and EHR data. Outcomes included platelet count, bleeding events, and corticosteroid exposure 3 to 6 months after initial treatment. Baseline platelet counts were lower in patients receiving early second-line therapy (10‒28 × 10^9^/L) versus those who did not (67 × 10^9^/L). Counts improved and bleeding events decreased from baseline in all treatment groups 3 to 6 months after the start of therapy. Among the very few patients for whom follow-up treatment data were available (*n* = 94), corticosteroid use was reduced during the 3- to 6-month follow-up period in patients who received early second-line therapy versus those who did not (39% vs 87%, *p* < 0.001). Early second-line treatment was prescribed for more severe cases of ITP and appeared to be associated with improved platelet counts and bleeding outcomes 3 to 6 months after initial therapy. Early second-line therapy also appeared to reduce corticosteroid use after 3 months, although the small number of patients with follow-up data on treatment precludes any substantive conclusions. Further research is needed to determine whether early second-line therapy has an effect on the long-term course of ITP.

**Supplementary Information:**

The online version contains supplementary material available at 10.1007/s00277-023-05289-0.

## Introduction

Primary immune thrombocytopenia (ITP) is an acquired autoimmune disorder characterized by a low platelet count (< 100 × 10^9^/L) due to impaired platelet production and accelerated platelet destruction as a result of anti-platelet autoantibodies and T-cell-mediated cytotoxicity [1]. The phenotype of ITP is extremely heterogeneous, with clinical features including bleeding being highly variable [2]. Accordingly, there are very few validated risk factors than can predict disease outcomes or response to therapies. Current recommendations vary depending on guidelines and countries, and the choice of ITP treatment remains principally dependent on single-arm studies, expert opinion, or patient preference rather than high-quality evidence from randomized controlled trials (RCTs) [3, 6].

First-line treatments for ITP have remained unchanged for decades, although there may be growing use of dexamethasone in preference to prednisone. These first-line treatments include corticosteroids, intravenous immunoglobulin (IVIG), anti-D immunoglobulin, and even platelet transfusions [7]. Second-line treatments are intended for long-term use, requiring high degrees of tolerability and safety, albeit at greater expense. Rituximab is an anti-CD20 monoclonal antibody sometimes used as a very early second-line treatment for ITP, with long-term remissions occurring in 21 to 26% of adults and children with ITP [8]. Thrombopoietin receptor agonists (TPO-RAs), which increase platelet production [9, 12], include eltrombopag, romiplostim, and avatrombopag. These are increasingly used as early second-line therapies, including in patients with newly diagnosed ITP. Other second-line treatments include fostamatinib, immunosuppressives, and splenectomy.

There is remarkably little data in adults on when and how often ITP will resolve, whether or not they receive standard treatments. One study from Austria suggested that 60% of patients get better within 3 years, but there is little data available regarding the first 3–6 months or even the first year of disease [5].

In this descriptive, non-interventional, retrospective, claims and health record-based cohort study, the therapeutic management of patients newly diagnosed with primary ITP was explored using real-world data from 2012 to 2019. Our objective was to assess the outcomes for adults and children with ITP who were prescribed early (within 3 months of initial treatment) second-line treatment(s) (i.e., eltrombopag, romiplostim, rituximab, splenectomy, immunosuppressive agents) compared with those who did not receive early second-line therapy. We hypothesized that early second-line treatment may be prescribed in patients with lower platelet counts and increased bleeding and that their use would lead to higher platelet counts, lower rates of bleeding, and less corticosteroid use during the 3 to 6 months after initial treatment.

## Methods

### Data source and ethics

#### Optum^®^ de-identified Electronic Health Record dataset

This was a descriptive, non-interventional, retrospective, cohort study of patients with primary ITP in the USA. The study used a secondary source of data, the Optum^®^ de-identified Electronic Health Record (EHR) dataset, which is a US-based, patient-level database that provides real-world data combining medical claims and health records for over 100 million patients from more than 150,000 providers at 2000 hospitals and over 7000 clinics [13]. The data contained in the database are from both outpatient and inpatient settings and include demographic characteristics, diagnoses, procedures, vital signs, medications prescribed and administered, laboratory test results, and notes recorded during routine clinical practice.

Optum’s EHR repository was chosen for this study because it provides laboratory test results such as platelet counts and because it contains longitudinal patient data with a number of follow-up years ranging from ≥ 1 year (~ 66%) to ≥ 5 years (~ 38%), enabling longitudinal analysis. Anonymized individual patient records from multiple sources of care are linked using a unique patient identifier.

The database is compliant with the Health Insurance Portability and Accountability Act (HIPAA) of 1996. The requirement for informed consent for use of protected health information was waived in accordance with the 1996 HIPAA because it was not practicable to request consent from all study patients for access to their medical records, and the risk to individuals’ privacy was determined to be minimal.

### Study population

Optum’s EHR repository was scanned for EHRs of patients with newly diagnosed primary ITP from January 1, 2012, to September 30, 2019 (identification period).

The *index diagnosis date* was defined as the date of diagnosis of ITP for a patient during the identification period, i.e., the first record in the database of a diagnosis code identifying a patient with ITP, primary thrombocytopenia, primary thrombocytopenia (unspecified), or other primary thrombocytopenia based on the International Classification of Diseases 9th Revision Clinical Modification (ICD-9-CM) or International Classification of Diseases 10th Revision Clinical Modification/Procedure Coding System (ICD-10-CM) (Supplemental Table1). The *index therapy date* was defined as the date of first ITP treatment within a period of 63 days (9 weeks) before or 365 days after index diagnosis date, recognizing that some newly diagnosed ITP patients are initiated on ITP therapy before or after their official diagnosis. Eligible ITP treatments are listed in Table 1.

**Table 1:** Qualifying ITP treatments

First-line treatments	Corticosteroids (prednisone, methylprednisolone, prednisolone, and/or dexamethasone)
	Intravenous immunoglobulin (IVIG)
	Platelet transfusion
Second-line treatments	Eltrombopag
	Romiplostim
	Rituximab
	Immunosuppressives (azathioprine or mycophenolate)
	Splenectomy

**Fig. 1:**
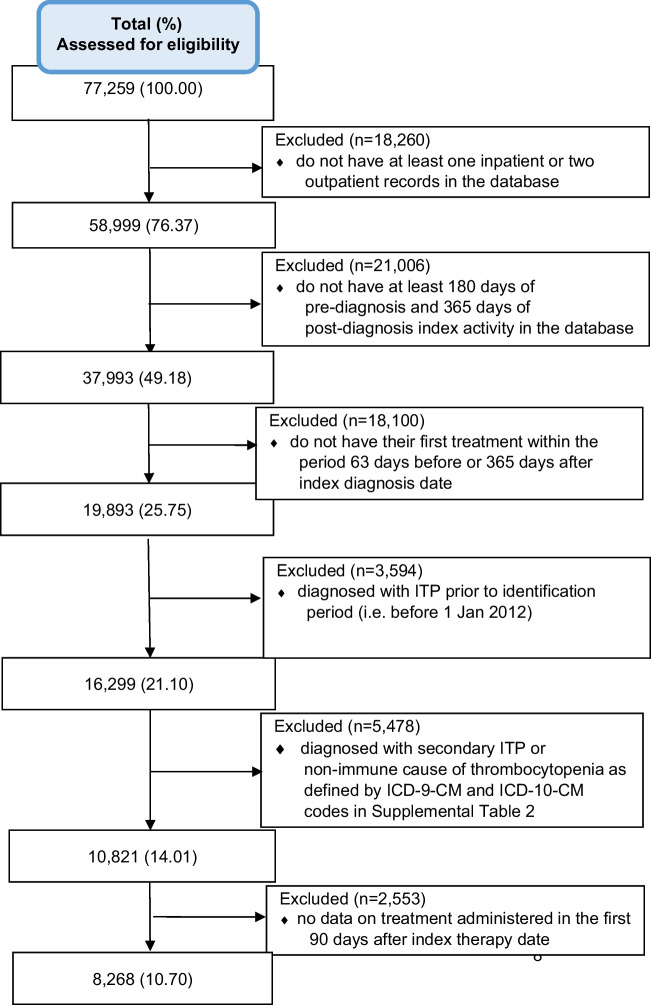
Sample attrition and cohort selection—attrition of patients with primary immune thrombocytopenia from Optum’s EHR repository for inclusion in the study cohort

The study period started 180 days prior to the start of the identification period to ensure that only incident ITP was captured and ended 365 days after the end of the identification period, with a *complete* study time period stretching from July 1, 2011, to September 30, 2020. The following predefined inclusion and exclusion criteria were used to select patients from Optum’s EHR repository for our study (see also attrition table; Fig. 1):

#### Inclusion criteria

Patients diagnosed with ITP, as defined by ICD-9-CM and ICD-10-CM codes in Supplemental Table 1, during the set identification period who received their first qualifying ITP therapy (Table 1) in the period from 63 days before to 365 days after the index diagnosis date

#### Exclusion criteria

Patients with less than one inpatient or less than two outpatient records (interaction types such as letter/email, telephone/online and swing bed were excluded)

Patients with less than 180 days of activity prior to or less than 365 days of activity following the index diagnosis date (activity being determined by looking at the earliest and the latest encounter in the database and defined as any diagnosis-, treatment-, or procedure-related event that is captured in the database)

Patients who did not receive their first ITP treatment in the period from 63 days before to 365 days after the index diagnosis date

Patients diagnosed with ITP prior to the specified identification period

Patients diagnosed with secondary ITP or non-immune causes of thrombocytopenia during the study period, as defined by ICD-9-CM and ICD-10-CM/PCS codes in Supplemental Table 2

Patients with no data on treatment received in the first 90 days after index therapy date

Patients were classified into treatment groups (“Eltrombopag,” “Romiplostim,” “Rituximab,” “Immunosuppressives,” and “Splenectomy”) according to the second-line treatment they received in the 90 days after the index therapy date (Table 2). Patients treated with multiple second-line treatments within 90 days after the index therapy date were assigned to the “Multiple second-line treatments” group. Patients assigned to these early second-line treatment groups may have received first-line treatment concomitantly. Those treated with only first-line treatments including corticosteroids, platelet transfusions, and/or intravenous immunoglobulin (IVIG) and with no second-line treatments within 90 days after the index therapy date were assigned to the “No second-line treatment” group.

**Table 2:** Early second-line treatment groups (as prescribed in the first 90 days of treatment)

1. Eltrombopag	Eltrombopag only second-line treatment^a^
2. Romiplostim	Romiplostim only second-line treatment^a^
3. Rituximab	Rituximab only second-line treatment^a^
4. Immunosuppressives	Either azathioprine or mycophenolate only second-line treatment^a^
5. Splenectomy	Splenectomy only second-line treatment^a^
6. Multiple second-line treatments	Two or more of eltrombopag, romiplostim, rituximab, azathioprine or mycophenolate, or splenectomy^a^
7. No second-line treatment^b^	No eltrombopag, romiplostim, rituximab, azathioprine, mycophenolate, or splenectomy

^a^First-line treatment may have been prescribed concomitantly

^b^The treatment group “no second-line treatment” consists of patients who were only prescribed first-line treatment during the first 90 days following treatment initiation (as listed in Table 1)

### Outcomes

We measured the following outcomes in the treatment groups defined in Table 2:

- Median platelet counts 91 to 180 days after the index therapy date
- Proportion of patients with at least one bleeding event 91 to 180 days after the index therapy date. The ICD-9-CM and ICD-10-CM/PCS codes for bleeding events were modified from Altomare et al. [14] (Supplemental Table 3). The proportion of patients with a bleeding event during the first 90 days after the index therapy date was also assessed as baseline.
- Proportion of patients prescribed any corticosteroids over the 91 to 180 days after the index therapy date

**Table 3:** Baseline characteristics according to the treatment group

Treatment group	Number of patients	Proportion of patients (%)	Median age at diagnosis (range), years	Baseline platelet count^a^ (IQR) (× 10^9^/L)	Proportion of patients with bleeding event(s) 0–90 days after index therapy date (%)
Eltrombopag	188	2.3	60 (4, 88)	24 (6–53)	13.8
Romiplostim	222	2.7	65 (4, 87)	28 (10‒52)	27.5
Rituximab	400	4.8	61 (2, 87)	19 (5‒54)	33.5
Immunosuppressives	23	0.3	62 (31, 85)	106 (93‒156)	17.4
Splenectomy	0	0	-	-	-
Multiple L2	108	1.3	61 (9, 87)	10 (5‒10)	43.5
No L2	7327	88.6	57 (1, 88)	67 (22‒111)	20.5

^a^Minimum platelet count from the start of observation (− 180 days from diagnosis) until the start of therapy (median of per-patient minimum). The overall study period is 180 days before and 365 days after ITP diagnosis

*IQR*, interquartile range taken as 25 percentile and 75 percentile of the baseline platelet counts; *L2*, second-line treatments

### Data analysis

All data were analyzed using Python version 3.7 and R version 3.4.3. We calculated descriptive statistics including patient demographics (age, gender, race, and ethnicity), treatment patterns, hospital visits, diagnoses including comorbidities, observations, and laboratory tests.

Baseline platelet count was calculated as the median of per-patient minimum platelet counts, as measured from the start of observation (− 180 days from diagnosis) until index therapy date. The median platelet count during the period of 91 to 180 days after treatment was calculated as the median of per-patient medians.

A chi-square test was used to compare the proportion of patients who used steroid during the 91–180-day follow-up period in the early second-line versus no early second-line treatment groups.

## Results

### Study population

Of 81,968,946 patients in Optum’s EHR repository, 77,259 (0.094%) had an ICD9/10 code for primary ITP (Supplemental Table 1) within the identification period. A total of 68,991 patients were excluded, resulting in a final cohort of 8268 patients with primary ITP, which constituted the final dataset on which the analyses were performed. Frequent reasons for exclusion were lack of activity in the database at least 180 days before and 365 days after the diagnosis index date and the absence of any qualifying ITP treatment (Table 1) within 63 days before or 365 days after the index diagnosis date (Fig. 1**)**. ITP treatment was initiated before the index diagnosis date in approximately one quarter of patients.

### Baseline characteristics

Overall, 58% of the cohort population was female. The majority were Caucasian (85%); 8% were African American, 2% Asian, and 5% Hispanic.

Baseline characteristics according to the treatment group are given in Table 3. Most ITP-treated patients (*n* = 7327; 88.6%) did not receive second-line therapy during the first 90 days of treatment. The most commonly used second-line agent in the first 90 days of treatment was rituximab (*n* = 400; 42.5%); 43.6% of patients received a TPO agent (romiplostim: 23.6% [*n* = 222] and eltrombopag: 20.0% [*n* = 188]), and 108 patients (11.5%) received multiple second-line therapies. Patients receiving multiple second-line treatments had the lowest baseline platelet count (10 × 10^9^/L), with those receiving rituximab at 19 × 10^9^/L, eltrombopag at 24 × 10^9^/L, and romiplostim at 28 × 10^9^/L having slightly higher counts. Patients on first-line treatment but not treated with early second-line therapy had a higher baseline platelet count (67 × 10^9^/L). The proportion of patients with at least one bleeding event within 90 days of the index therapy date was highest in the multiple second-line treatment group.

### Platelet counts

**Fig. 2:**
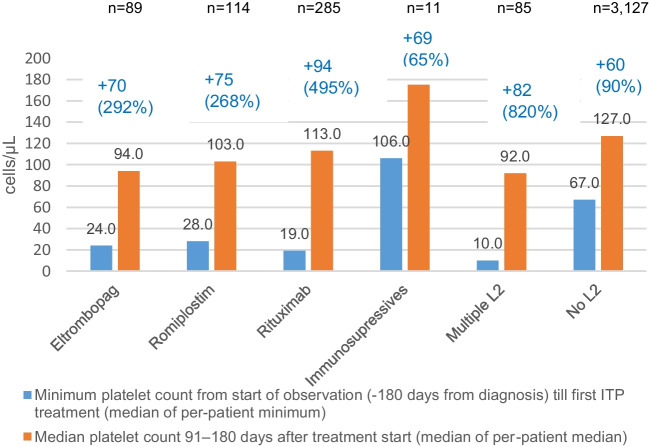
Platelet counts at baseline and 91 to 180 days after index therapy date. Baseline defined as minimum platelet count from the start of observation (− 180 days from diagnosis) until the start of therapy (median of per-patient minimum). n, number of patients with platelet count data 91–180 days after treatment start (index therapy date). Only those patients with platelet count values available were included in the analysis (subset of patients included in each treatment group). No cases of splenectomy were reported; therefore, this category was not included in the graph. L2, second-line treatments

Compared with baseline, the median platelet count between day 91 and day 365 increased in all treatment groups (Fig. 2).

### Bleeding events

**Fig. 3:**
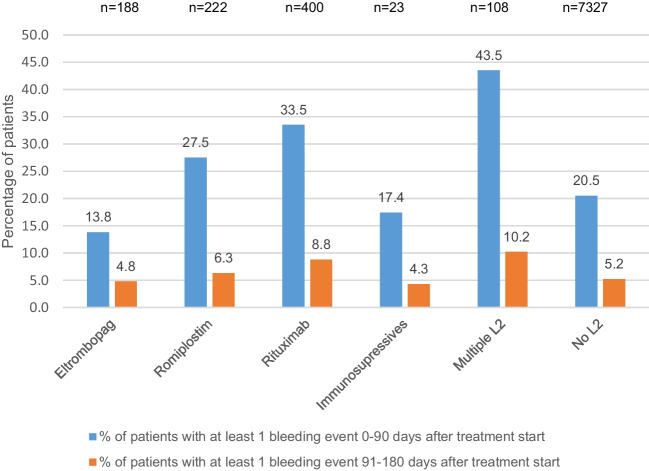
Proportion of patients with bleeding event(s) at baseline and 91 to 180 days after index therapy date. n, number of patients with bleeding event data available 91 to 180 days after treatment start (index therapy date). No cases of splenectomy were reported; therefore, this category was not included in the graph. L2, second-line treatments

Compared with baseline, the proportion of patients with a bleeding event 91 to 180 days after the index therapy date decreased in all treatment groups (Fig. 3). The largest reduction in the proportion of patients with bleeding events was observed in the group receiving multiple second-line therapies.

### Corticosteroid use

Information on corticosteroid usage between day 91 and day 180 was only available for approximately 1% of the study population. However, among those for whom it was available, only 13 of 33 patients (39%) treated with second-line therapy early (in the first 3 months of treatment) received steroids in the following 3 to 6 months. In contrast, 53 of 61 patients (87%) who did not receive second-line therapy in the first 3 months were treated with steroids during the following 3 months (*p* < 0.001).

## Discussion

This non-interventional, retrospective, real-world evidence study assessed the outcomes of 8268 patients with primary ITP who met the study criteria and did or did not receive early second-line therapy. While about 8/9 of eligible patients with ITP received only first-line therapy (predominantly corticosteroids) within the first 3 months after treatment initiation, approximatively 11% of patients received “early” second-line treatment with eltrombopag, romiplostim, rituximab, immunosuppressives, or a combination of these agents, with or without first-line therapy. In this analysis, no patient underwent early splenectomy during this 3-month period.

In many patients, ITP treatment was initiated before the index diagnosis date. This presumably reflects, at least in part, the urgent need for immediate treatment of thrombocytopenia despite delays in confirmation of the ITP diagnosis by a specialist, as well-described in the I-WISh study [15]. Furthermore, in some cases, treatments such as corticosteroids might have been started for another indication, which may also partly explain the high platelet count observed at baseline in this treatment group (67 × 10^9^/L).

Compared with baseline, platelet counts improved and bleeding events decreased in all treatment groups by 3 to 6 months after the index therapy date, irrespective of whether early second-line therapy was used or not. However, the relative platelet increase was lower in patients who did not receive early second-line therapy (+ 60 [90%]) compared with the increase observed in other treatment groups (+ 75‒94 [268‒495%] with TPO-RAs or rituximab and + 82 [820%] with multiple second-line therapies). The benefits observed in patients receiving multiple second-line therapies was especially striking given the lower baseline platelet counts in this population. These excellent outcomes highlight that the early use of these therapies appeared to be highly appropriate.

Moreover, in the very limited number of patients with available data, early second-line therapy was associated with substantially less corticosteroid use between 3 and 6 months (less than 2 of 5 patients vs 7 of 8 patients who did not receive early second-line treatment, *p* < 0.001). The improved outcomes in patients who received early second-line treatment are all the more interesting given that these patients tended to have more severe disease at presentation, as evidenced by lower platelet counts and more bleeding events at baseline (Table 3). The American Society of Hematology guidelines [4] and an international consensus report [5] recommend avoidance of prolonged corticosteroid exposure. Our findings suggest the potential value of early second-line therapy as a means of reducing steroid use, although the strength of this hypothesis is limited by the very small amount of data available.

### Limitations

Our study has several limitations. As in all analyses using EHR data, there is a potential for coding errors, possibly leading to misclassification bias [16]. We were also missing data for outcomes of interest. For a number of reasons (listed in Fig. 1), many patients had to be excluded so that the analysis cohort reflects only about 1 in 10 of those identified as having ITP during the time period in question. Among the eligible patients, missing platelet counts may explain why the median nadir platelet count was higher than expected in the no second-line and immunosuppressive-treatment groups (Table 3). In addition, we are missing a considerable amount of follow-up data on treatment. Data were available for treatment months 3 to 6 in only about 1% of the overall cohort. This data gap may be the result of a failure to record treatment details, for instance for patients who may see a doctor out of the captured network, such as a primary care provider. Another important possibility, however, is that a substantial number of adults with newly diagnosed ITP improve and stop seeing their hematologist. Surprisingly little is known about the course of adults with ITP, in particular how often and when it improves sufficiently to discontinue treatment. Finally, owing to limited follow-up time, our analysis cannot address whether early use of second-line therapy ameliorates the long-term disease course. Further research is needed to address this and other related questions.

## Conclusion

Our findings suggest that early second-line therapy in patients with ITP is associated with improvement in platelet counts and reduced rates of bleeding, as demonstrated in the more severe cases. If the limited treatment data between days 91 and 180 is reflective of the overall group, then early second-line therapy may be associated with reduced corticosteroid exposure between 3 and 6 months after initiation of therapy. Additional research is needed to confirm these findings, particularly the reduction in subsequent corticosteroid use, and to better understand the course of ITP in adults beyond 3 months from initiation of treatment.

## Supplementary Information

Below is the link to the electronic supplementary material.

Supplementary file1 (DOCX 48 KB)

## Data Availability

Data for this study was made available through a third-party license from Optum^®^ EHR, a commercial data provider in the USA. Further release of the dataset is not possible due to a data use agreement.
