# Supplementary material for: Early initiation of second-line therapy in primary immune thrombocytopenia: insights from real-world evidence
Source: Ann Hematol. 2023 Jun 10;102(8):2051–8. doi: 10.1007/s00277-023-05289-0 (PMC10345059; doi:10.1007/s00277-023-05289-0)
Supplement: Supplementary file 1 — Supplementary file1 (DOCX 48 KB) [file 277_2023_5289_MOESM1_ESM.docx]

# Supplementary material

**Title**

**Early initiation of second line therapy in primary immune thrombocytopenia:** **insights from real-world evidence**

Target journal: **Annals of Hematology**

**Authors**: Adam Cuker^1^, Brian Buckley^2^, Marie-Catherine Mousseau^2^, Aditya Anand Barve^2^, Jens Haenig^3^, James B. Bussel^4^

1. Department of Medicine and Department of Pathology and Laboratory Medicine, Perelman School of Medicine, University of Pennsylvania, Philadelphia, PA, USA
2. Novartis Ireland Limited, Dublin, Ireland
3. Novartis Pharma AG, Basel, Switzerland
4. Department of Pediatrics, Weill Cornell Medicine, New York, NY, USA

**Corresponding author**

Adam Cuker, MD, MS

Hospital of the University of Pennsylvania

3 Dulles, 3400 Spruce Street

Philadelphia, Pennsylvania

[Adam.Cuker@uphs.upenn.edu](mailto:Adam.Cuker@uphs.upenn.edu)

Telephone: +1 (215) 615-6555

Fax: +1 (215) 615-6596

###### Table 1 International Classification of Diseases codes used to identify patients with immune thrombocytopenia

| **Codes** | **Disease** |
| --- | --- |
| ICD10CM D69.3 | Immune thrombocytopenic purpura |
| ICD10CM D69.4 | Other primary thrombocytopenia |
| ICD10CM D69.49 | Other primary thrombocytopenia |
| ICD9CM 287.3 | Primary thrombocytopenia |
| ICD9CM 287.30 | Primary thrombocytopenia, unspecified |
| ICD9CM 287.31 | Immune thrombocytopenic purpura |
| ICD9CM 287.39 | Other primary thrombocytopenia |

###### Table 2 International Classification of Diseases codes used to identify patients with secondary immune thrombocytopenia or non-immune causes of thrombocytopenia

| **Vocabulary** | **Code** | **Description** |
| --- | --- | --- |
| ICD9CM | 42 | Human immunodeficiency virus [HIV] disease |
| ICD9CM | 284 | Aplastic anemia and other bone marrow failure syndromes |
| ICD9CM | 2830 | Autoimmune hemolytic anemias |
| ICD9CM | 2839 | Acquired hemolytic anemia, unspecified |
| ICD9CM | 2840 | Constitutional aplastic anemia |
| ICD9CM | 2841 | Pancytopenia |
| ICD9CM | 2842 | Myelophthisis |
| ICD9CM | 2848 | Other specified aplastic anemias |
| ICD9CM | 2849 | Aplastic anemia, unspecified |
| ICD9CM | 2866 | Defibrination syndrome |
| ICD9CM | 2894 | Hypersplenism |
| ICD9CM | 4466 | Thrombotic microangiopathy |
| ICD9CM | 5711 | Acute alcoholic hepatitis |
| ICD9CM | 7051 | Acute hepatitis C without mention of hepatic coma |
| ICD9CM | 7100 | Systemic lupus erythematosus |
| ICD9CM | 7953 | Human immunodeficiency virus, type 2 [HIV-2] |
| ICD9CM | 23872 | Low grade myelodysplastic syndrome lesions |
| ICD9CM | 23873 | High grade myelodysplastic syndrome lesions |
| ICD9CM | 23874 | Myelodysplastic syndrome with 5q deletion |
| ICD9CM | 23875 | Myelodysplastic syndrome, unspecified |
| ICD9CM | 27901 | Selective IgA immunodeficiency |
| ICD9CM | 28311 | Hemolytic-uremic syndrome |
| ICD9CM | 28409 | Other constitutional aplastic anemia |
| ICD9CM | 28411 | Antineoplastic chemotherapy induced pancytopenia |
| ICD9CM | 28412 | Other drug-induced pancytopenia |
| ICD9CM | 28419 | Other pancytopenia |
| ICD9CM | 28489 | Other specified aplastic anemias |
| ICD9CM | 28733 | Congenital and hereditary thrombocytopenic purpura |
| ICD9CM | 28749 | Other secondary thrombocytopenia |
| ICD9CM | 28984 | Heparin-induced thrombocytopenia (HIT) |
| ICD9CM | 79571 | Nonspecific serologic evidence of human immunodeficiency virus [HIV] |
| ICD10CM | B1710 | Acute hepatitis C without hepatic coma |
| ICD10CM | B1711 | Acute hepatitis C with hepatic coma |
| ICD10CM | B20 | Human immunodeficiency virus [HIV] disease |
| ICD10CM | B9735 | Human immunodeficiency virus, type 2 [HIV 2] as the cause of diseases classified elsewhere |
| ICD10CM | D460 | Refractory anemia without ring sideroblasts, so stated |
| ICD10CM | D461 | Refractory anemia with ring sideroblasts |
| ICD10CM | D4620 | Refractory anemia with excess of blasts, unspecified |
| ICD10CM | D4621 | Refractory anemia with excess of blasts 1 |
| ICD10CM | D4622 | Refractory anemia with excess of blasts 2 |
| ICD10CM | D469 | Myelodysplastic syndrome, unspecified |
| ICD10CM | D46A | Refractory cytopenia with multilineage dysplasia |
| ICD10CM | D46B | Refractory cytopenia with multilineage dysplasia and ring sideroblasts |
| ICD10CM | D46C | Myelodysplastic syndrome with isolated del(5q) chromosomal abnormality |
| ICD10CM | D591 | Other autoimmune hemolytic anemias |
| ICD10CM | D593 | Hemolytic-uremic syndrome |
| ICD10CM | D599 | Acquired hemolytic anemia, unspecified |
| ICD10CM | D61 | Other aplastic anemias and other bone marrow failure syndromes |
| ICD10 | D61 | Other aplastic anemias |
| ICD10 | D610 | Constitutional aplastic anemia |
| ICD10CM | D610 | Constitutional aplastic anemia |
| ICD10CM | D6109 | Other constitutional aplastic anemia |
| ICD10CM | D611 | Drug-induced aplastic anemia |
| ICD10 | D611 | Drug-induced aplastic anemia |
| ICD10CM | D612 | Aplastic anemia due to other external agents |
| ICD10 | D612 | Aplastic anemia due to other external agents |
| ICD10 | D613 | Idiopathic aplastic anemia |
| ICD10CM | D613 | Idiopathic aplastic anemia |
| ICD10CM | D618 | Other specified aplastic anemias and other bone marrow failure syndromes |
| ICD10 | D618 | Other specified aplastic anemias |
| ICD10CM | D6181 | Pancytopenia |
| ICD10CM | D61810 | Antineoplastic chemotherapy induced pancytopenia |
| ICD10CM | D61811 | Other drug-induced pancytopenia |
| ICD10CM | D61818 | Other pancytopenia |
| ICD10CM | D6182 | Myelophthisis |
| ICD10CM | D6189 | Other specified aplastic anemias and other bone marrow failure syndromes |
| ICD10 | D619 | Aplastic anemia, unspecified |
| ICD10CM | D619 | Aplastic anemia, unspecified |
| ICD10CM | D65 | Disseminated intravascular coagulation [defibrination syndrome] |
| ICD10CM | D6861 | Antiphospholipid syndrome |
| ICD10CM | D6862 | Lupus anticoagulant syndrome |
| ICD10CM | D6942 | Congenital and hereditary thrombocytopenia purpura |
| ICD10CM | D695 | Secondary thrombocytopenia |
| ICD10CM | D6951 | Posttransfusion purpura |
| ICD10CM | D6959 | Other secondary thrombocytopenia |
| ICD10CM | D731 | Hypersplenism |
| ICD10CM | D7582 | Heparin induced thrombocytopenia (HIT) |
| ICD10CM | D802 | Selective deficiency of immunoglobulin A [IgA] |
| ICD10CM | K7010 | Alcoholic hepatitis without ascites |
| ICD10CM | M311 | Thrombotic microangiopathy |
| ICD10CM | M3210 | Systemic lupus erythematosus, organ or system involvement unspecified |
| ICD10CM | R75 | Inconclusive laboratory evidence of human immunodeficiency virus [HIV] |
| ICD9CM | V08 | Asymptomatic human immunodeficiency virus [HIV] infection status |
| ICD9CM | V6544 | Human immunodeficiency virus (HIV) counseling |
| ICD10CM | Z21 | Asymptomatic human immunodeficiency virus [HIV] infection status |
| ICD10CM | Z717 | Human immunodeficiency virus [HIV] counseling |

###### Table 3 International Classification of Diseases codes used to identify patients with bleeding events. Adapted from Altomare et al. (2016).

| **Vocabulary** | **Code** | **Description** |
| --- | --- | --- |
| ICD9CM | 0786 | Hemorrhagic nephrosonephritis |
| ICD9CM | 2463 | Hemorrhage and infarction of thyroid |
| ICD9CM | 2872 | Other nonthrombocytopenic purpuras |
| ICD9CM | 2878 | Other specified hemorrhagic conditions |
| ICD9CM | 2879 | Unspecified hemorrhagic conditions |
| ICD9CM | 36043 | Hemophthalmos, except current injury |
| ICD9CM | 36243 | Hemorrhagic detachment of retinal pigment epithelium |
| ICD9CM | 36281 | Retinal hemorrhage |
| ICD9CM | 36361 | Choroidal hemorrhage, unspecified |
| ICD9CM | 36362 | Expulsive choroidal hemorrhage |
| ICD9CM | 36372 | Hemorrhagic choroidal detachment |
| ICD9CM | 37272 | Conjunctival hemorrhage |
| ICD9CM | 37481 | Hemorrhage of eyelid |
| ICD9CM | 37632 | Orbital hemorrhage |
| ICD9CM | 37742 | Hemorrhage in optic nerve sheaths |
| ICD9CM | 3786 | Mechanical strabismus |
| ICD9CM | 37860 | Mechanical strabismus, unspecified |
| ICD9CM | 37861 | Brown’s (tendon) sheath syndrome |
| ICD9CM | 37862 | Mechanical strabismus from other musculofascial disorders |
| ICD9CM | 37863 | Limited duction associated with other conditions |
| ICD9CM | 37923 | Vitreous hemorrhage |
| ICD9CM | 38031 | Hematoma of auricle or pinna |
| ICD9CM | 4230 | Hemopericardium |
| ICD9CM | 430 | Subarachnoid hemorrhage |
| ICD9CM | 431 | Intracerebral hemorrhage |
| ICD9CM | 4320 | Nontraumatic extradural hemorrhage |
| ICD9CM | 4321 | Subdural hemorrhage |
| ICD9CM | 4329 | Unspecified intracranial hemorrhage |
| ICD9CM | 4560 | Esophageal varices with bleeding |
| ICD9CM | 45620 | Esophageal varices in diseases classified elsewhere, with bleeding |
| ICD9CM | 4590 | Hemorrhage, unspecified |
| ICD9CM | 4786 | Edema of larynx |
| ICD9CM | 53021 | Ulcer of esophagus with bleeding |
| ICD9CM | 53082 | Esophageal hemorrhage |
| ICD9CM | 53501 | Acute gastritis, with hemorrhage |
| ICD9CM | 53511 | Atrophic gastritis, with hemorrhage |
| ICD9CM | 53521 | Gastric mucosal hypertrophy, with hemorrhage |
| ICD9CM | 53541 | Other specified gastritis, with hemorrhage |
| ICD9CM | 53551 | Unspecified gastritis and gastroduodenitis, with hemorrhage |
| ICD9CM | 53561 | Duodenitis, with hemorrhage |
| ICD9CM | 53571 | Eosinophilic gastritis, with hemorrhage |
| ICD9CM | 56202 | Diverticulosis of small intestine with hemorrhage |
| ICD9CM | 56203 | Diverticulitis of small intestine with hemorrhage |
| ICD9CM | 56212 | Diverticulosis of colon with hemorrhage |
| ICD9CM | 56213 | Diverticulitis of colon with hemorrhage |
| ICD9CM | 56881 | Hemoperitoneum (nontraumatic) |
| ICD9CM | 5693 | Hemorrhage of rectum and anus |
| ICD9CM | 5780 | Hematemesis |
| ICD9CM | 5781 | Blood in stool |
| ICD9CM | 5789 | Hemorrhage of gastrointestinal tract, unspecified |
| ICD9CM | 5967 | Hemorrhage into bladder wall |
| ICD9CM | 59970 | Hematuria, unspecified |
| ICD9CM | 59971 | Gross hematuria |
| ICD9CM | 59972 | Microscopic hematuria |
| ICD9CM | 6021 | Congestion or hemorrhage of prostate |
| ICD9CM | 60882 | Hematospermia |
| ICD9CM | 6207 | Hematoma of broad ligament |
| ICD9CM | 6214 | Hematometra |
| ICD9CM | 6245 | Hematoma of vulva |
| ICD9CM | 6263 | Puberty bleeding |
| ICD9CM | 6265 | Ovulation bleeding |
| ICD9CM | 6266 | Metrorrhagia |
| ICD9CM | 6267 | Postcoital bleeding |
| ICD9CM | 6268 | Other disorders of menstruation and other abnormal bleeding from female genital tract |
| ICD9CM | 6269 | Unspecified disorders of menstruation and other abnormal bleeding from female genital tract |
| ICD9CM | 6270 | Premenopausal menorrhagia |
| ICD9CM | 6271 | Postmenopausal bleeding |
| ICD9CM | 6290 | Hematocele, female, not elsewhere classified |
| ICD9CM | 63410 | Spontaneous abortion, complicated by delayed or excessive hemorrhage, unspecified |
| ICD9CM | 63411 | Spontaneous abortion, complicated by delayed or excessive hemorrhage, incomplete |
| ICD9CM | 63412 | Spontaneous abortion, complicated by delayed or excessive hemorrhage, complete |
| ICD9CM | 64080 | Other specified hemorrhage in early pregnancy, unspecified as to episode of care or not applicable |
| ICD9CM | 64081 | Other specified hemorrhage in early pregnancy, delivered, with or without mention of antepartum condition |
| ICD9CM | 64083 | Other specified hemorrhage in early pregnancy, antepartum condition or complication |
| ICD9CM | 64090 | Unspecified hemorrhage in early pregnancy, unspecified as to episode of care or not applicable |
| ICD9CM | 64091 | Unspecified hemorrhage in early pregnancy, delivered, with or without mention of antepartum condition |
| ICD9CM | 64093 | Unspecified hemorrhage in early pregnancy, antepartum condition or complication |
| ICD9CM | 64110 | Hemorrhage from placenta previa, unspecified as to episode of care or not applicable |
| ICD9CM | 64111 | Hemorrhage from placenta previa, delivered, with or without mention of antepartum condition |
| ICD9CM | 64113 | Hemorrhage from placenta previa, antepartum condition or complication |
| ICD9CM | 64130 | Antepartum hemorrhage associated with coagulation defects, unspecified as to episode of care or not applicable |
| ICD9CM | 64131 | Antepartum hemorrhage associated with coagulation defects, delivered, with or without mention of antepartum condition |
| ICD9CM | 64133 | Antepartum hemorrhage associated with coagulation defects, antepartum condition or complication |
| ICD9CM | 64180 | Other antepartum hemorrhage, unspecified as to episode of care or not applicable |
| ICD9CM | 64181 | Other antepartum hemorrhage, delivered, with or without mention of antepartum condition |
| ICD9CM | 64183 | Other antepartum hemorrhage, antepartum condition or complication |
| ICD9CM | 64190 | Unspecified antepartum hemorrhage, unspecified as to episode of care or not applicable |
| ICD9CM | 64191 | Unspecified antepartum hemorrhage, delivered, with or without mention of antepartum condition |
| ICD9CM | 64193 | Unspecified antepartum hemorrhage, antepartum condition or complication |
| ICD9CM | 65600 | Fetal-maternal hemorrhage, unspecified as to episode of care or not applicable |
| ICD9CM | 65601 | Fetal-maternal hemorrhage, delivered, with or without mention of antepartum condition |
| ICD9CM | 65603 | Fetal-maternal hemorrhage, antepartum condition or complication |
| ICD9CM | 66570 | Pelvic hematoma, unspecified as to episode of care or not applicable |
| ICD9CM | 66571 | Pelvic hematoma, delivered, with or without mention of antepartum condition |
| ICD9CM | 66572 | Pelvic hematoma, delivered with mention of postpartum complication |
| ICD9CM | 66574 | Pelvic hematoma, postpartum condition or complication |
| ICD9CM | 66600 | Third-stage postpartum hemorrhage, unspecified as to episode of care or not applicable |
| ICD9CM | 66602 | Third-stage postpartum hemorrhage, delivered, with mention of postpartum complication |
| ICD9CM | 66604 | Third-stage postpartum hemorrhage, postpartum condition or complication |
| ICD9CM | 66610 | Other immediate postpartum hemorrhage, unspecified as to episode of care or not applicable |
| ICD9CM | 66612 | Other immediate postpartum hemorrhage, delivered, with mention of postpartum complication |
| ICD9CM | 66614 | Other immediate postpartum hemorrhage, postpartum condition or complication |
| ICD9CM | 66620 | Delayed and secondary postpartum hemorrhage, unspecified as to episode of care or not applicable |
| ICD9CM | 66622 | Delayed and secondary postpartum hemorrhage, delivered, with mention of postpartum complication |
| ICD9CM | 66624 | Delayed and secondary postpartum hemorrhage, postpartum condition or complication |
| ICD9CM | 71910 | Hemarthrosis, site unspecified |
| ICD9CM | 71911 | Hemarthrosis, shoulder region |
| ICD9CM | 71912 | Hemarthrosis, upper arm |
| ICD9CM | 71913 | Hemarthrosis, forearm |
| ICD9CM | 71914 | Hemarthrosis, hand |
| ICD9CM | 71915 | Hemarthrosis, pelvic region and thigh |
| ICD9CM | 71916 | Hemarthrosis, lower leg |
| ICD9CM | 71917 | Hemarthrosis, ankle and foot |
| ICD9CM | 71918 | Hemarthrosis, other specified sites |
| ICD9CM | 71919 | Hemarthrosis, multiple sites |
| ICD9CM | 7621 | Other forms of placental separation and hemorrhage affecting fetus or newborn |
| ICD9CM | 76711 | Epicranial subaponeurotic hemorrhage (massive) |
| ICD9CM | 7703 | Pulmonary hemorrhage |
| ICD9CM | 77210 | Intraventricular hemorrhage unspecified grade |
| ICD9CM | 77211 | Intraventricular hemorrhage, grade I |
| ICD9CM | 77212 | Intraventricular hemorrhage, grade II |
| ICD9CM | 77213 | Intraventricular hemorrhage, grade III |
| ICD9CM | 77214 | Intraventricular hemorrhage, grade IV |
| ICD9CM | 7722 | Subarachnoid hemorrhage of fetus or newborn |
| ICD9CM | 7723 | Umbilical hemorrhage after birth |
| ICD9CM | 7724 | Gastrointestinal hemorrhage of fetus or newborn |
| ICD9CM | 7726 | Cutaneous hemorrhage of fetus or newborn |
| ICD9CM | 7728 | Other specified hemorrhage of fetus or newborn |
| ICD9CM | 7729 | Unspecified hemorrhage of newborn |
| ICD9CM | 7773 | Hematemesis and melena of newborn due to swallowed maternal blood |
| ICD9CM | 7786 | Congenital hydrocele |
| ICD9CM | 7827 | Spontaneous ecchymoses |
| ICD9CM | 7847 | Epistaxis |
| ICD9CM | 7848 | Hemorrhage from throat |
| ICD9CM | 78630 | Hemoptysis, unspecified |
| ICD9CM | 78639 | Other hemoptysis |
| ICD9CM | 7912 | Hemoglobinuria |
| ICD9CM | 8786 | Open wound of vagina, without mention of complication |
| ICD9CM | 9786 | Poisoning by pertussis vaccine, including combinations with a pertussis component |
| ICD9CM | E8786 | Removal of other organ (partial) (total) causing abnormal patient reaction, or later complication, without mention of misadventure at time of operation |
| ICD10CM | A985 | Hemorrhagic fever with renal syndrome |
| ICD10CM | D692 | Other nonthrombocytopenic purpura |
| ICD10CM | D698 | Other specified hemorrhagic conditions |
| ICD10CM | D699 | Hemorrhagic condition, unspecified |
| ICD10CM | E0789 | Other specified disorders of thyroid |
| ICD10CM | H0289 | Other specified disorders of eyelid |
| ICD10CM | H05239 | Hemorrhage of unspecified orbit |
| ICD10CM | H1133 | Conjunctival hemorrhage, bilateral |
| ICD10CM | H31309 | Unspecified choroidal hemorrhage, unspecified eye |
| ICD10CM | H31319 | Expulsive choroidal hemorrhage, unspecified eye |
| ICD10CM | H31419 | Hemorrhagic choroidal detachment, unspecified eye |
| ICD10CM | H3560 | Retinal hemorrhage, unspecified eye |
| ICD10CM | H35739 | Hemorrhagic detachment of retinal pigment epithelium, unspecified eye |
| ICD10CM | H4313 | Vitreous hemorrhage, bilateral |
| ICD10CM | H44819 | Hemophthalmos, unspecified eye |
| ICD10CM | H47029 | Hemorrhage in optic nerve sheath, unspecified eye |
| ICD10CM | H61129 | Hematoma of pinna, unspecified ear |
| ICD10CM | I312 | Hemopericardium, not elsewhere classified |
| ICD10CM | I609 | Nontraumatic subarachnoid hemorrhage, unspecified |
| ICD10CM | I619 | Nontraumatic intracerebral hemorrhage, unspecified |
| ICD10CM | I6200 | Nontraumatic subdural hemorrhage, unspecified |
| ICD10CM | I621 | Nontraumatic extradural hemorrhage |
| ICD10CM | I629 | Nontraumatic intracranial hemorrhage, unspecified |
| ICD10CM | I8501 | Esophageal varices with bleeding |
| ICD10CM | I8511 | Secondary esophageal varices with bleeding |
| ICD10CM | K2901 | Acute gastritis with bleeding |
| ICD10CM | K2921 | Alcoholic gastritis with bleeding |
| ICD10CM | K2941 | Chronic atrophic gastritis with bleeding |
| ICD10CM | K2951 | Unspecified chronic gastritis with bleeding |
| ICD10CM | K2961 | Other gastritis with bleeding |
| ICD10CM | K2971 | Gastritis, unspecified, with bleeding |
| ICD10CM | K2981 | Duodenitis with bleeding |
| ICD10CM | K2991 | Gastroduodenitis, unspecified, with bleeding |
| ICD10CM | K5281 | Eosinophilic gastritis or gastroenteritis |
| ICD10CM | K5660 | Unspecified intestinal obstruction |
| ICD10CM | K56600 | Partial intestinal obstruction, unspecified as to cause |
| ICD10CM | K56601 | Complete intestinal obstruction, unspecified as to cause |
| ICD10CM | K56609 | Unspecified intestinal obstruction, unspecified as to partial versus complete obstruction |
| ICD10CM | K5711 | Diverticulosis of small intestine without perforation or abscess with bleeding |
| ICD10CM | K5713 | Diverticulitis of small intestine without perforation or abscess with bleeding |
| ICD10CM | K5731 | Diverticulosis of large intestine without perforation or abscess with bleeding |
| ICD10CM | K5733 | Diverticulitis of large intestine without perforation or abscess with bleeding |
| ICD10CM | K625 | Hemorrhage of anus and rectum |
| ICD10CM | K661 | Hemoperitoneum |
| ICD10CM | K920 | Hematemesis |
| ICD10CM | K921 | Melena |
| ICD10CM | K922 | Gastrointestinal hemorrhage, unspecified |
| ICD10CM | M2500 | Hemarthrosis, unspecified joint |
| ICD10CM | M25019 | Hemarthrosis, unspecified shoulder |
| ICD10CM | M25029 | Hemarthrosis, unspecified elbow |
| ICD10CM | M25039 | Hemarthrosis, unspecified wrist |
| ICD10CM | M25049 | Hemarthrosis, unspecified hand |
| ICD10CM | M25059 | Hemarthrosis, unspecified hip |
| ICD10CM | M25069 | Hemarthrosis, unspecified knee |
| ICD10CM | M25073 | Hemarthrosis, unspecified ankle |
| ICD10CM | M25076 | Hemarthrosis, unspecified foot |
| ICD10CM | M2508 | Hemarthrosis, other specified site |
| ICD10CM | N3289 | Other specified disorders of bladder |
| ICD10CM | N421 | Congestion and hemorrhage of prostate |
| ICD10CM | N837 | Hematoma of broad ligament |
| ICD10CM | N857 | Hematometra |
| ICD10CM | N9089 | Other specified noninflammatory disorders of vulva and perineum |
| ICD10CM | N921 | Excessive and frequent menstruation with irregular cycle |
| ICD10CM | N922 | Excessive menstruation at puberty |
| ICD10CM | N923 | Ovulation bleeding |
| ICD10CM | N924 | Excessive bleeding in the premenopausal period |
| ICD10CM | N925 | Other specified irregular menstruation |
| ICD10CM | N926 | Irregular menstruation, unspecified |
| ICD10CM | N930 | Postcoital and contact bleeding |
| ICD10CM | N938 | Other specified abnormal uterine and vaginal bleeding |
| ICD10CM | N939 | Abnormal uterine and vaginal bleeding, unspecified |
| ICD10CM | N9489 | Other specified conditions associated with female genital organs and menstrual cycle |
| ICD10CM | N950 | Postmenopausal bleeding |
| ICD10CM | O031 | Delayed or excessive hemorrhage following incomplete spontaneous abortion |
| ICD10CM | O036 | Delayed or excessive hemorrhage following complete or unspecified spontaneous abortion |
| ICD10CM | O046 | Delayed or excessive hemorrhage following (induced) termination of pregnancy |
| ICD10CM | O071 | Delayed or excessive hemorrhage following failed attempted termination of pregnancy |
| ICD10CM | O081 | Delayed or excessive hemorrhage following ectopic and molar pregnancy |
| ICD10CM | O208 | Other hemorrhage in early pregnancy |
| ICD10CM | O209 | Hemorrhage in early pregnancy, unspecified |
| ICD10CM | O46001 | Antepartum hemorrhage with coagulation defect, unspecified, first trimester |
| ICD10CM | O46002 | Antepartum hemorrhage with coagulation defect, unspecified, second trimester |
| ICD10CM | O46003 | Antepartum hemorrhage with coagulation defect, unspecified, third trimester |
| ICD10CM | O46009 | Antepartum hemorrhage with coagulation defect, unspecified, unspecified trimester |
| ICD10CM | O46011 | Antepartum hemorrhage with afibrinogenemia, first trimester |
| ICD10CM | O46012 | Antepartum hemorrhage with afibrinogenemia, second trimester |
| ICD10CM | O46013 | Antepartum hemorrhage with afibrinogenemia, third trimester |
| ICD10CM | O46019 | Antepartum hemorrhage with afibrinogenemia, unspecified trimester |
| ICD10CM | O46021 | Antepartum hemorrhage with disseminated intravascular coagulation, first trimester |
| ICD10CM | O46022 | Antepartum hemorrhage with disseminated intravascular coagulation, second trimester |
| ICD10CM | O46023 | Antepartum hemorrhage with disseminated intravascular coagulation, third trimester |
| ICD10CM | O46029 | Antepartum hemorrhage with disseminated intravascular coagulation, unspecified trimester |
| ICD10CM | O46091 | Antepartum hemorrhage with other coagulation defect, first trimester |
| ICD10CM | O46092 | Antepartum hemorrhage with other coagulation defect, second trimester |
| ICD10CM | O46093 | Antepartum hemorrhage with other coagulation defect, third trimester |
| ICD10CM | O46099 | Antepartum hemorrhage with other coagulation defect, unspecified trimester |
| ICD10CM | O468x1 | Other antepartum hemorrhage, first trimester |
| ICD10CM | O468X1 | Other antepartum hemorrhage, first trimester |
| ICD10CM | O468x2 | Other antepartum hemorrhage, second trimester |
| ICD10CM | O468X2 | Other antepartum hemorrhage, second trimester |
| ICD10CM | O468x3 | Other antepartum hemorrhage, third trimester |
| ICD10CM | O468X3 | Other antepartum hemorrhage, third trimester |
| ICD10CM | O468x9 | Other antepartum hemorrhage, unspecified trimester |
| ICD10CM | O468X9 | Other antepartum hemorrhage, unspecified trimester |
| ICD10CM | O4690 | Antepartum hemorrhage, unspecified, unspecified trimester |
| ICD10CM | O4691 | Antepartum hemorrhage, unspecified, first trimester |
| ICD10CM | O4692 | Antepartum hemorrhage, unspecified, second trimester |
| ICD10CM | O4693 | Antepartum hemorrhage, unspecified, third trimester |
| ICD10CM | O670 | Intrapartum hemorrhage with coagulation defect |
| ICD10CM | O678 | Other intrapartum hemorrhage |
| ICD10CM | O679 | Intrapartum hemorrhage, unspecified |
| ICD10CM | O717 | Obstetric hematoma of pelvis |
| ICD10CM | O720 | Third-stage hemorrhage |
| ICD10CM | O721 | Other immediate postpartum hemorrhage |
| ICD10CM | O722 | Delayed and secondary postpartum hemorrhage |
| ICD10CM | P261 | Massive pulmonary hemorrhage originating in the perinatal period |
| ICD10CM | P268 | Other pulmonary hemorrhages originating in the perinatal period |
| ICD10CM | P518 | Other umbilical hemorrhages of newborn |
| ICD10CM | P520 | Intraventricular (nontraumatic) hemorrhage, grade 1, of newborn |
| ICD10CM | P521 | Intraventricular (nontraumatic) hemorrhage, grade 2, of newborn |
| ICD10CM | P5221 | Intraventricular (nontraumatic) hemorrhage, grade 3, of newborn |
| ICD10CM | P5222 | Intraventricular (nontraumatic) hemorrhage, grade 4, of newborn |
| ICD10CM | P523 | Unspecified intraventricular (nontraumatic) hemorrhage of newborn |
| ICD10CM | P525 | Subarachnoid (nontraumatic) hemorrhage of newborn |
| ICD10CM | P548 | Other specified neonatal hemorrhages |
| ICD10CM | R040 | Epistaxis |
| ICD10CM | R041 | Hemorrhage from throat |
| ICD10CM | R042 | Hemoptysis |
| ICD10CM | R0481 | Acute idiopathic pulmonary hemorrhage in infants |
| ICD10CM | R0489 | Hemorrhage from other sites in respiratory passages |
| ICD10CM | R049 | Hemorrhage from respiratory passages, unspecified |
| ICD10CM | R233 | Spontaneous ecchymoses |
| ICD10CM | R310 | Gross hematuria |
| ICD10CM | R311 | Benign essential microscopic hematuria |
| ICD10CM | R312 | Other microscopic hematuria |
| ICD10CM | R3121 | Asymptomatic microscopic hematuria |
| ICD10CM | R3129 | Other microscopic hematuria |
| ICD10CM | R319 | Hematuria, unspecified |
| ICD10CM | R361 | Hematospermia |
| ICD10CM | R58 | Hemorrhage, not elsewhere classified |
| ICD10CM | R823 | Hemoglobinuria |

Altomare I, Cetin K, Wetten S, Wasser JS (2016) Rate of bleeding-related episodes in adult patients with primary immune thrombocytopenia: a retrospective cohort study using a large administrative medical claims database in the US. Clinical epidemiology 8:231-239. doi:10.2147/clep.s105888
